# Spatiotemporal Feature Learning for Daily-Life Cough Detection Using FMCW Radar

**DOI:** 10.3390/bioengineering12101112

**Published:** 2025-10-15

**Authors:** Saihu Lu, Yuhan Liu, Guangqiang He, Zhongrui Bai, Zhenfeng Li, Pang Wu, Xianxiang Chen, Lidong Du, Peng Wang, Zhen Fang

**Affiliations:** 1 Aerospace Information Research Institute, Chinese Academy of Sciences (AIRCAS), Beijing 100094, China; lusaihu20@mails.ucas.ac.cn (S.L.); liuyuhan243@mails.ucas.ac.cn (Y.L.); zhongrui.bai@sjtu.edu.cn (Z.B.); lizhenfeng@aircas.ac.cn (Z.L.); wupang@aircas.ac.cn (P.W.); chenxx@aircas.ac.cn (X.C.); lddu@mail.ie.ac.cn (L.D.); 2 School of Electronic, Electrical and Communication Engineering, University of Chinese Academy of Sciences, Beijing 100049, China; 3 National Graduate School for Elite Engineers, Shandong University, Jinan 250100, China; hgq@mail.sdu.edu.cn; 4 School of Electronic Information, Aerospace Information Technology University, Jinan 250299, China; 5 School of Electronic Information and Electrical Engineering, Shanghai Jiao Tong University, Shanghai 200240, China

**Keywords:** cough detection, FMCW radar, deep learning, health care

## Abstract

Cough is a key symptom reflecting respiratory health, with its frequency and pattern providing valuable insights into disease progression and clinical management. Objective and reliable cough detection systems are therefore of broad significance for healthcare and remote monitoring. However, existing algorithms often struggle to jointly model spatial and temporal information, limiting their robustness in real-world applications. To address this issue, we propose a cough recognition framework based on frequency-modulated continuous-wave (FMCW) radar, integrating a deep convolutional neural network (CNN) with a Self-Attention mechanism. The CNN extracts spatial features from range-Doppler maps, while Self-Attention captures temporal dependencies, and effective data augmentation strategies enhance generalization by simulating position variations and masking local dependencies. To rigorously evaluate practicality, we collected a large-scale radar dataset covering diverse positions, orientations, and activities. Experimental results demonstrate that, under subject-independent five-fold cross-validation, the proposed model achieved a mean F1-score of 0.974±0.016 and an accuracy of 99.05±0.55 %, further supported by high precision of 98.77±1.05 %, recall of 96.07±2.16 %, and specificity of 99.73±0.23 %. These results confirm that our method is not only robust in realistic scenarios but also provides a practical pathway toward continuous, non-invasive, and privacy-preserving respiratory health monitoring in both clinical and telehealth applications.

## 1. Introduction

Cough is a common clinical symptom associated with a range of respiratory diseases, including asthma, chronic bronchitis, and chronic obstructive pulmonary disease, and can serve as an important indicator of disease exacerbation [1]. In recent years, cough frequency has increasingly been recognized as a quantifiable parameter for monitoring respiratory conditions and has also been employed to evaluate recovery following exacerbations [2]. The medical literature further emphasizes the need for developing automated, objective, and reliable systems for detecting cough events [3].

Several objective methods for cough monitoring have been proposed. Audio-based systems dominate the field: Drugman et al. [4] compared microphone types for cough detection, Liu et al. [5] applied deep neural networks to spectrogram features, while Vhaduri et al. [6] and Wang et al. [7] developed smartphone- and CNN-based systems for noisy and clinical environments, respectively. Despite their effectiveness, such approaches remain vulnerable to environmental noise and raise privacy concerns in sensitive settings [6,7]. Wearable systems provide direct physiological sensing: Saeed et al. [8] introduced a bias-mitigated CNN-LSTM, and Otoshi et al. [9] achieved 92% sensitivity with a dual-sensor patch. Yet, wearables require continuous skin contact, leading to hygiene and compliance issues [10,11]. In contrast, radar-based sensing is contactless, privacy-preserving, and resilient to noise, making it well-suited for long-term deployment [12]. In recent years, artificial intelligence (AI) and deep learning, particularly convolutional neural networks (CNNs), have gained increasing attention in biomedical research for their ability to extract hierarchical features and model complex patterns [13,14]. These methods have been successfully applied to neurological studies [15,16]. Their widespread adoption highlights their potential to improve diagnostic accuracy and support personalized healthcare.

Recent studies have demonstrated the potential of radar technology for cough monitoring [17,18,19,20,21,22]. In [19,20,21,22], different radar systems and signal processing techniques were employed to extract spectrograms or time-domain representations corresponding to cough events. In [17,18], radar signal preprocessing methods were combined with data-driven classifiers to perform cough recognition, showing encouraging results. Nevertheless, these approaches have not fully exploited the spatiotemporal information available in radar data, including range, velocity, and temporal features. This highlights a critical gap: the lack of a unified framework that holistically captures the full spatiotemporal dynamics of a cough event. A cough is not merely a velocity event, but a complex motion involving specific chest and torso displacements (range) that evolve over a short duration (time). By neglecting either the spatial or temporal dimension, previous works risk confusing coughs with other short-term movements, limiting their robustness in complex daily-life scenarios. Therefore, a model that jointly learns from range, velocity, and their temporal evolution is essential for achieving high-accuracy, reliable cough detection.

In this study, we investigate cough recognition using millimeter-wave frequency-modulated continuous-wave (FMCW) radar. A deep learning framework is proposed, termed ST-CoughNet (Spatiotemporal Cough Network), which combines a deep CNN with a Self-Attention mechanism. By jointly modeling range, velocity, and temporal information captured by FMCW radar, the proposed framework aims to achieve accurate and reliable cough detection. Specifically, the ResNet module extracts spatial features from individual range-Doppler maps (RDMs), capturing range and velocity information, while the Self-Attention module encodes temporal dependencies across the RDM sequence. Our framework allows the model to learn rich spatiotemporal representations and improve classification performance.

To further enhance robustness, we introduce a radar-specific data augmentation strategy that increases data diversity without compromising the physical integrity of radar measurements. This approach enables the model to capture more diverse and informative patterns, improving generalization under real-world conditions.

For dataset collection, variability in user positions and body postures encountered in daily living environments was considered. A large-scale dataset was constructed under diverse positions, postures, and orientations to ensure experimental credibility and ecological validity. The proposed model’s effectiveness was rigorously validated through a subject-independent evaluation protocol and a series of ablation studies, demonstrating its high accuracy and robustness in cough recognition.

The main contributions of this work are summarized as follows:

1. We propose ST-CoughNet, a framework for cough recognition leveraging millimeter-wave FMCW radar. By exploiting the inherent range, velocity, and temporal information embedded in radar signals, the model effectively captures spatiotemporal characteristics of human activities, thereby achieving competitive performance in cough recognition.
2. We devise a data augmentation strategy specifically tailored to the radar data representation used in this study. This strategy increases data diversity while preserving the physical meaning of radar signals, improving the model’s generalization capability and robustness.
3. We construct a comprehensive mmWave radar dataset for cough recognition. Under a subject-independent evaluation protocol, the ST-CoughNet model combined with the proposed augmentation method achieves an accuracy of 99.05% and an F1-score of 0.974 in cough recognition. Ablation studies further confirmed the contribution of each component and the effectiveness of the data augmentation strategy.

## 2. Related Work

### 2.1. Radar Cough Detection

Numerous studies have explored the potential of radar technology for cough detection, with several reporting encouraging preliminary results. In this section, we review relevant works in this field.

Some studies process radar data using specialized algorithms to extract cough-related signals, demonstrating the feasibility of radar-based cough monitoring. For instance, Lin et al. [19] employed a multi-frequency continuous-wave (MFCW) agile radar, where ensemble empirical mode decomposition (EEMD) and principal component analysis (PCA) were applied to cross-correlation results to mitigate the nonlinear effects of respiratory harmonics or intermodulation tones, enabling the acquisition of cough time-domain signals. Han and Kawon et al. [20,21] utilized FMCW radar and applied the body motion artifact cancellation (BMAC) technique to suppress motion artifacts, followed by a phase demodulation method to obtain spectrograms of cough events. Hämäläinen et al. [22] adopted an ultra-wideband (UWB) impulse radar to capture cough time-domain signals, demonstrating that UWB signals can remotely detect subtle chest movements associated with coughs. Collectively, these studies indicate that radar can effectively detect coughs and retrieve corresponding radar signatures.

Chuma et al. [17] employed a K-band continuous-wave Doppler radar combined with a CNN architecture for cough detection, utilizing velocity and temporal information but not incorporating range information representing the distance between the target and the radar, achieving an accuracy of 86.5% with AlexNet. This approach leveraged velocity and temporal information but did not utilize range information, leading to incomplete use of spatiotemporal data. Zhang et al. [23] proposed an FMCW radar-based approach for detecting sleep apnea, a common respiratory disorder, and demonstrated high accuracy in event-level segmentation of respiratory events. Yao et al. [24] employed FMCW radar for fall detection across diverse scenarios by extracting three types of information from radar signals: the range–velocity map, the range–horizontal angle map, and the range–vertical angle map. Collectively, these studies indicate that radar spatiotemporal signals contain rich and discriminative information, which can be systematically leveraged for effective feature extraction. Jugović et al. [18] applied FMCW radar and CNNs to classify movement types (rest, motion, and cough) from FMCW radar phase data collected from 10 supine participants. Their preprocessing algorithm segmented radar data into different range bins, after which the magnitude-phase coherence (MPC) algorithm was used to select the optimal bin. The time-varying radar signal intensity from this bin was then input to a CNN for classification. While these methods yielded promising results, they underutilized spatiotemporal information: only a single range bin was exploited, despite other bins containing relevant target information, and velocity features were not incorporated.

### 2.2. Data Augmentation

Data augmentation has been widely adopted in deep learning to address challenges of limited training samples or class imbalance [21]. In this section, we introduce several data augmentation techniques commonly used in image recognition and discuss their adaptation to radar data representation.

Image translation is a classical augmentation technique. By shifting images, this method artificially increases data diversity and can improve learning performance [25]. Shijie et al. [26] applied various augmentation techniques to the CIFAR-10 dataset and a subset of ImageNet, conducting a comparative analysis of their effects and combinations across different training scales. The results indicated that image translation can effectively enhance classification accuracy.

Random erasing is a more recent data augmentation method designed for training CNNs. During training, a rectangular region within an image is randomly selected, and its pixels are replaced with random values. This generates training images with varying degrees of occlusion, encouraging the network to focus on previously overlooked local features rather than overemphasizing dominant features. Consequently, random erasing can mitigate overfitting and improve model robustness to occlusions. The method requires no additional parameter learning, is easy to implement, and can be integrated into most CNN-based recognition models [27]. Moreover, it achieved improvements in object detection and person re-identification tasks, indicating its potential applicability across diverse recognition scenarios.

Although these approaches originated in image recognition, our study processes radar signals into image-like representations (e.g., RDM). Therefore, applying such augmentation strategies is effective for radar-based tasks, as they enhance generalization, alleviate overfitting, and improve robustness under diverse real-world conditions.

## 3. Method

### 3.1. Data Preprocessing

In FMCW radar systems, the transmitted signal frequency increases linearly over time, commonly referred to as a linear frequency modulated pulse (chirp). The transmitting antenna sends this pulse, which is reflected by an object and received by the radar’s receiving antenna. The mixer combines the received signal (RX) with the transmitted signal (TX) to generate an intermediate frequency (IF) signal.

To extract range and velocity information of the target, the FMCW radar transmits multiple chirps continuously. Consequently, the mixer outputs multiple IF signals, which are organized into an M×N matrix, where *M* represents the number of consecutively transmitted chirps and *N* denotes the number of ADC samples per chirp. For a radar with *R* receiving antennas, *R* such M×N matrices are generated.

Each chirp in the IF signal matrix undergoes a Fast Fourier Transform (FFT) transformation along the sampling dimension, referred to as range-FFT, to obtain distance information. The resulting spectrum exhibits peaks corresponding to objects at specific distances, effectively separating the radar data into different range bins. After performing the range-FFT, each received signal $f_{i}\left(n\right)$ from each antenna is normalized by subtracting the mean across all samples to suppress static objects:

(1) $${\tilde{f}}_{i}\left(n\right)=f_{i}\left(n\right)-\frac{1}{M}\sum_{j=1}^{M}f_{j}\left(n\right),$$

where ${\tilde{f}}_{i}\left(n\right)$ denotes the clutter-removed signal of the *i*-th chirp, $f_{i}\left(n\right)$ represents the IF signal, i=1,2,…,M is the chirp index, and n=1,2,…,N is the ADC sampling index within each chirp.

To remove range bins with negligible information, we calculated the average signal across all antennas and then took the modulus of the result. Additionally, averaging across multiple receiving antennas helps reduce noise and improve accuracy. The range bin with the strongest reflected signal is considered the primary range bin. In this study, we extracted 48 range bins centered on this primary bin, which are assumed to contain the relevant activity information of the subjects.

**Figure 1:**
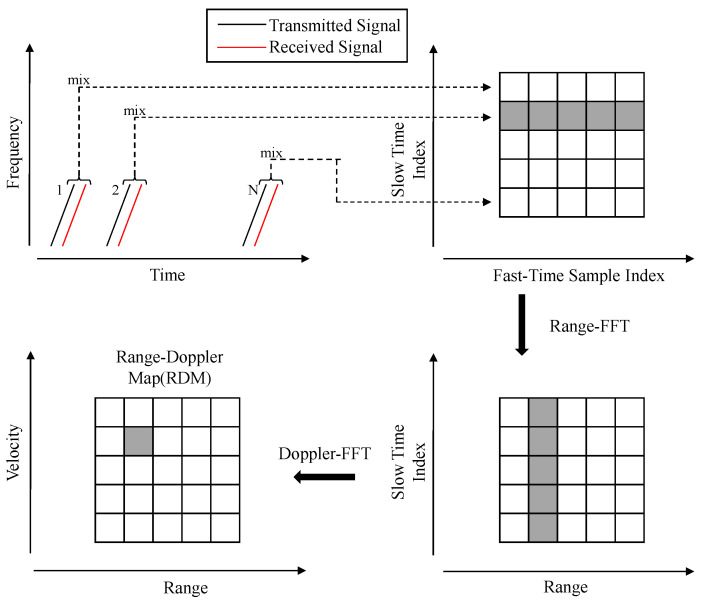
The signal processing pipeline for generating an range-Doppler map (RDM) from FMCW radar signals. The process begins (**top-left**) with a sequence of transmitted and received chirps in the time-frequency domain. After mixing and sampling, these signals form a raw data matrix (**top-right**), with axes representing the Slow Time Index and Fast Time Index. A Range-FFT is applied along each row (fast-time dimension) to resolve the target’s range (**bottom-right**). Subsequently, a Doppler-FFT is applied along each column (slow-time dimension) to resolve the target’s velocity, producing the final RDM (**bottom-left**) where the axes represent range and velocity.

To obtain the velocity component at each distance, an FFT is applied along the chirp dimension of each range bin, referred to as Doppler-FFT. Processing the radar signal through range-FFT and Doppler-FFT produces the RDM [28]. In the RDM, negative velocities indicate objects moving toward the radar, while positive velocities correspond to objects moving away. The RDM thus reflects the movement of different parts of the target. The range-FFT and Doppler-FFT processing pipeline is illustrated in Figure 1.

In the experimental setup of this study, the raw radar data are complex-valued arrays with shape (NUM_CHIRPS, NUM_TX, NUM_RX, NUM_ADC_SAMPLES). The specific parameters are as follows:

- NUM_CHIRPS: 1500, denoting the number of chirps.
- NUM_TX: 1, indicating the number of transmitting antennas.
- NUM_RX: 4, indicating the number of receiving antennas.
- NUM_ADC_SAMPLES: 108, corresponding to the number of sampling points per chirp.

As described above, the radar data were acquired at a frame rate of 500 Hz, with one chirp per frame, resulting in a total of 1500 chirps recorded over 3 s. To compute RDMs, a sliding-window Doppler-FFT was applied within each range bin, where the window length was set to 125 chirps and the step size to 40 chirps. This procedure produces RDM sequences with dimensions of 35×1×48×125 . Here, 35 denotes the number of short-time frames generated by the sliding window, 1 denotes the number of channels, 48 corresponds to the number of selected range bins, and 125 represents the length of the Doppler spectrum.

Since the RDM is complex-valued, we compute its magnitude and then average across multiple receiving antennas to improve signal reliability. Furthermore, a logarithmic transformation is applied to the RDM to enhance weak reflected components, such as those caused by cough events, thereby facilitating the model in capturing discriminative information.

### 3.2. Feature Extraction Module

In this experiment, human movements in front of the radar produce echo signals, which are subsequently processed into a sequence of RDMs. Each RDM characterizes the target’s range and Doppler velocity information within a short time interval. Consequently, the sequence of RDMs reflects the temporal evolution of the target’s range–velocity distribution relative to the radar. Accurate identification of cough events therefore, requires the extraction of both spatial and temporal features from the RDM sequences.

**Figure 2:**
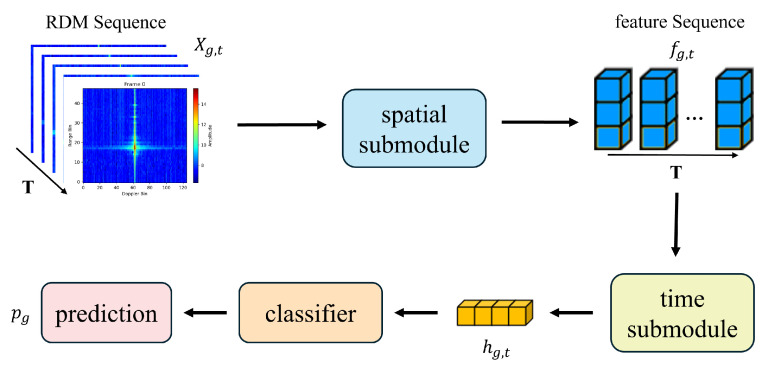
Architecture of the proposed ST-CoughNet model. The model takes a sequence of T RDMs, denoted as $X_{g,t}$ , as input. The enlarged RDM example visualizes the range–velocity distribution of a signal, where the axes represent Doppler velocity and range, and the color bar indicates signal power intensity. The processing pipeline consists of (1) a spatial submodule (ResNet) that extracts a feature sequence $f_{g,t}$ from each RDM; (2) a temporal submodule (Self-Attention) that captures dependencies across the sequence to produce an aggregated feature vector $h_{g,t}$ ; and (3) a final classifier that outputs the prediction probability $p_{g}$ .

Specifically, spatial features describe the range–velocity distribution within an individual RDM, while temporal features capture the dynamic variations in this distribution over time [29,30]. In recent years, ResNet has been widely adopted in image recognition tasks [31,32], and Self-Attention mechanisms [33] have demonstrated strong capability in modeling temporal dependencies. Motivated by these advances, we propose a spatiotemporal feature extraction framework consisting of a spatial submodule based on ResNet and a temporal submodule employing Self-Attention. The spatial submodule extracts range–velocity features from individual RDMs, while the temporal submodule learns the correlations across the entire RDM sequence. The fused spatiotemporal features are then passed to a downstream classification module, which outputs the recognition results. The overall structure of the proposed model is illustrated in Figure 2.

**Figure 3:**
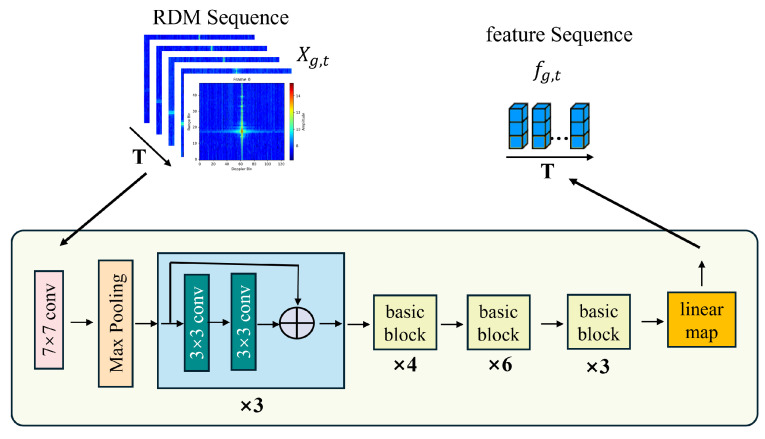
The spatial feature extraction submodule, which employs a modified ResNet-34 architecture. The module processes each RDM from the input sequence $X_{g,t}$ to extract a high-level spatial feature vector. To adapt the network for feature extraction, the original fully connected classification layer of ResNet-34 is replaced with a linear map that projects the features into the output sequence $f_{g,t}$ .

Figure 3 illustrates the spatial submodule of the proposed model. As shown, this module employs ResNet-34 to extract high-level spatial features from each RDM in the input sequence, producing the spatial feature sequence as follows:

(2) $$f_{g,t}=G_{\theta_{\mathrm{ResNet}\text{-}34}}\;\left(X_{g,t}\right),\;t=1,2,\ldots ,T,$$

where $X_{g,t}\in R^{1\times 48\times 125}$ denotes the *t*-th range-Doppler map in the sequence T=35 of subject *g*, and $\theta_{\mathrm{ResNet}\text{-}34}$ represents the parameters of the modified ResNet-34 network. The extracted spatial feature for each frame is $f_{g,t}\in R^{256}$ , and the full sequence of features is expressed as $f_{g,t}=\left\{f_{g,1},f_{g,2},\ldots ,f_{g,T}\right\}\in R^{T\times 256}$ .

The rationale for adopting ResNet-34 lies in its residual learning mechanism, which enables the network to preserve low-level structural information while progressively capturing more abstract spatial patterns. Compared with shallower CNNs, ResNet-34 avoids gradient vanishing and overfitting problems, allowing the model to generalize effectively on radar data.

In our case, the input radar data are represented as RDMs, which inherently encode the joint distribution of target distance and relative velocity. The convolutional filters of ResNet-34 are well-suited for extracting localized patterns such as spectral ridges, motion trajectories, and micro-Doppler signatures from these 2D maps. After layer-by-layer abstraction, the network produces feature embeddings that highlight spatial correlations related to human respiratory motion and cough events.

By combining these spatial embeddings with the subsequent temporal submodule, the framework not only captures static geometric characteristics but also encodes dynamic variations across frames. This integration ensures that both the physical properties of radar echoes and their temporal evolution are preserved in the learned representation. Ultimately, the spatial submodule serves as the foundation for robust detection by transforming raw RDMs into compact yet discriminative features aligned with the underlying physical phenomena.

**Figure 4:**
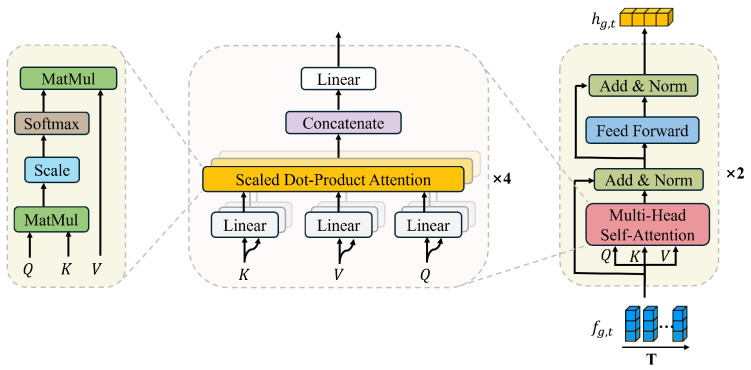
The temporal modeling submodule, designed to capture relationships across the time dimension of the feature sequence $f_{g,t}$ . The module uses two stacked layers, each employing a multi-head Self-Attention mechanism to weigh the importance of different features over time, followed by a feed-forward network. The expanded views detail how the multi-head attention is constructed from several parallel scaled dot-product attention units. The module outputs a temporally enhanced feature sequence $h_{g,t}$ .

Figure 4 illustrates the temporal submodule of the proposed model. This module takes the sequence of spatial features $f_{g,t}$ as input and captures temporal dependencies through two stacked layers, each comprising a multi-head Self-Attention mechanism with four attention heads, followed by a position-wise feed-forward network, residual connections, and layer normalization. This design enables the model to jointly attend to information from multiple temporal perspectives while maintaining stable optimization. The learned temporal features are then concatenated with the corresponding spatial features to form a unified spatiotemporal representation of the RDM sequence, expressed as

(3) $$h_{g,t}=G_{\theta_{\mathrm{SA}}}\;\left(f_{g,1},f_{g,2},\ldots ,f_{g,T}\right),\;t=1,2,\ldots ,T,$$

where $\theta_{\mathrm{SA}}$ denotes the learnable parameters of the Self-Attention module. For each subject *g*, the input sequence $\left\{f_{g,1},f_{g,2},\ldots ,f_{g,T}\right\}\in R^{T\times 256}$ is transformed into a temporally enhanced representation $\left\{h_{g,1},h_{g,2},\ldots ,h_{g,T}\right\}$ , where each $h_{g,t}\in R^{256}$ encodes contextual dependencies across time.

The rationale for using Self-Attention lies in its ability to dynamically assign weights to different frames, enabling the model to emphasize frames containing salient respiratory or cough-induced variations while suppressing irrelevant or noisy segments. Compared with recurrent architectures, the Self-Attention mechanism can capture both short- and long-range dependencies without suffering from gradient vanishing, which is particularly beneficial given the temporal irregularities of cough events.

By integrating the spatial submodule with the temporal submodule, the framework not only preserves fine-grained spatial cues—such as Doppler shifts and spectral ridges—but also models their temporal evolution across frames. This ensures that both static information (e.g., subject posture) and dynamic information (e.g., transient motion patterns associated with coughing) are effectively represented. Ultimately, the fusion of spatial and temporal features allows the model to construct a compact yet discriminative representation of radar echoes, enhancing its robustness in contactless cough recognition.

**Figure 5:**
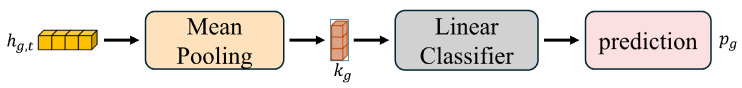
The downstream classification head for final cough prediction. This module takes the temporally aware feature sequence $h_{g,t}$ as input. The processing involves two stages: First, a temporal mean pooling layer aggregates the entire sequence along its time axis into a single, fixed-size feature vector $k_{g}$ . Second, this aggregated vector is passed to a linear classifier, which computes the final prediction probability vector $p_{g}$ for the ‘cough’ and ‘non-cough’ classes.

Figure 5 illustrates the downstream classifier of the proposed model. The extracted spatiotemporal features $h_{g}^{1,2,\ldots ,T}$ are first processed through a mean pooling layer along the temporal dimension to obtain a compact representation $k_{g}$ , which is subsequently fed into a linear classifier to generate the final prediction results $p_{g}^{0,1}$ :

(4) $$k_{g}=G_{\mathrm{MP}}\;\left(h_{g,1},h_{g,2},\ldots ,h_{g,T}\right)=\frac{1}{T}\sum_{t=1}^{T}h_{g,t},$$

where $G_{\mathrm{MP}}$ denotes temporal mean pooling applied across all *T* frames. Here, each $h_{g,t}\in R^{256}$ represents the temporal feature at frame *t*, and the resulting vector $k_{g}\in R^{256}$ is the aggregated representation for subject *g*, which serves as the input to the classification layer.

(5) $$p_{g}=G_{\theta_{\mathrm{LC}}}\left(k_{g}\right),$$

where $G_{\theta_{\mathrm{LC}}}$ denotes the linear classifier parameterized by $\theta_{\mathrm{LC}}$ . The resulting probability vector $p_{g}=\left[p_{g}^{\left(0\right)},p_{g}^{\left(1\right)}\right]\in R^{2}$ represents the predicted likelihood that the sample *g* belongs to class c=0 (non-cough) or c=1 (cough).

The objective of this study is to correctly identify the target’s behavior as coughing or non-coughing. To guide the training, we adopt the cross-entropy loss:

(6) $$L_{\mathrm{CE}}=-\frac{1}{N}\sum_{g=1}^{N}\sum_{c=0}^{1}y_{g}^{\left(c\right)}\log\left(p_{g}^{\left(c\right)}\right),$$

where $L_{\mathrm{CE}}$ denotes the cross-entropy loss computed over a batch of *N* samples. Here, $y_{g}^{\left(c\right)}\in \left\{0,1\right\}$ is the one-hot ground truth label indicating whether the *g*-th sample belongs to class c∈{0,1} (non-cough or cough), and $p_{g}^{\left(c\right)}\in \left[0,1\right]$ is the corresponding predicted probability obtained from the classifier output $p_{g}=\left[p_{g}^{\left(0\right)},p_{g}^{\left(1\right)}\right]\in R^{2}$ .

### 3.3. Data Augmentation

Data augmentation is widely employed in deep learning to help models learn richer representations from limited data [34]. In this study, we extend two commonly used image-based augmentation methods, image translation and random erasing, to make them applicable to RDM sequences.

**Figure 6:**
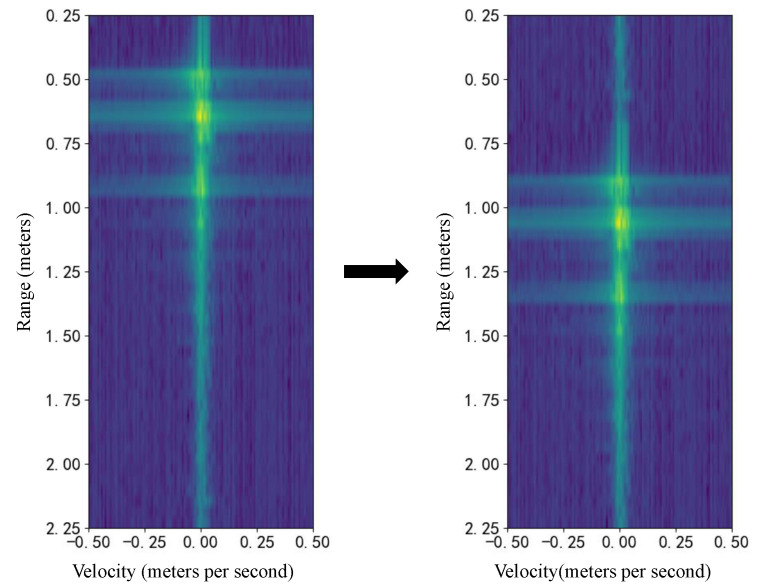
The image translation data augmentation technique applied to an RDM. The original RDM (**left**) is randomly shifted along the vertical (range) axis to create the augmented sample (**right**), simulating a change in target distance. The RDM axes show target range (meters) versus velocity (meters per second), with signal power indicated by color.

Specifically, image translation is applied along the distance dimension of the RDM, simulating target signals at different distances and thereby increasing the diversity of the training data. This approach can enhance the model’s robustness to variations in target position and facilitate the capture of additional feature information. An example of a single RDM after translation is shown in Figure 6. Since radar data consist of sequences of RDMs, it is important to apply the same translation consistently across all RDMs in a sequence to preserve temporal relationships; that is, each RDM should be shifted in the same direction by the same number of pixels.

**Figure 7:**
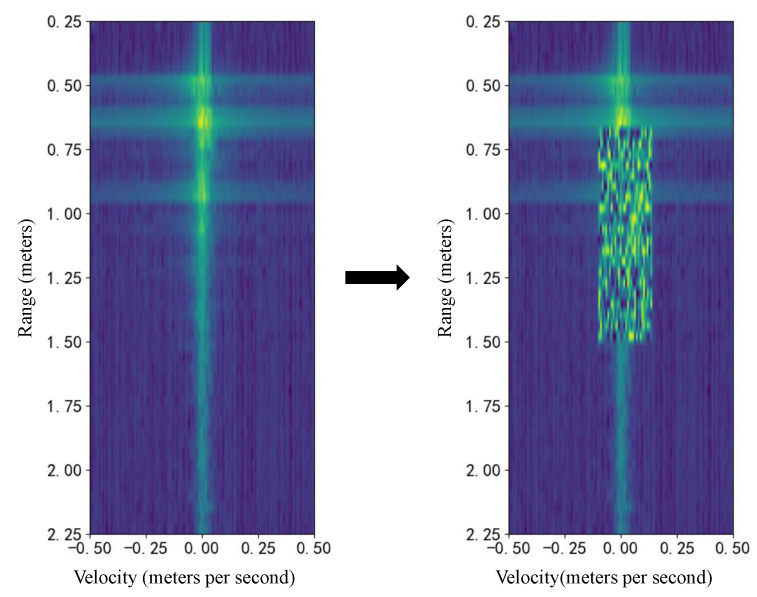
The random erasing data augmentation technique applied to an RDM. A randomly selected rectangular region of the original RDM (**left**) is masked with noise to create the augmented sample (**right**). The RDM axes show target range (meters) versus velocity (meters per second), with signal power indicated by color.

Random erasing [27] is used to mask a randomly selected rectangular region within an RDM by filling it with random values within the original data range, with the area and aspect ratio of the region sampled within predefined ranges. This technique prevents the model from over-relying on specific regions of the RDM, encouraging it to learn more generalized feature representations. Consequently, it can help mitigate overfitting and improve the model’s generalization capability. Similar to image translation, the masking operation should be applied consistently across all RDMs in a sequence to avoid disrupting temporal feature extraction. An example of a single RDM after random erasing is shown in Figure 7.

### 3.4. Experimental Setup

Due to the lack of publicly available datasets, we validated the proposed method on a dataset collected in our laboratory. Specifically, we used the IWR6843ISK millimeter-wave radar and the DCA1000 real-time data capture board from Texas Instruments (Dallas, TX, USA).

For the radar configuration, the chirp repetition frequency was set to 500 Hz, and a total of 3 s of data was collected, resulting in 1500 chirps, with 108 sampling points per chirp. Detailed radar parameters are listed in Table 1. Under these settings, the radar achieves a range resolution of approximately 0.042 m and a velocity resolution of approximately 0.008 m per second. The system consists of one transmitting antenna and four receiving antennas.

**Figure 8:**
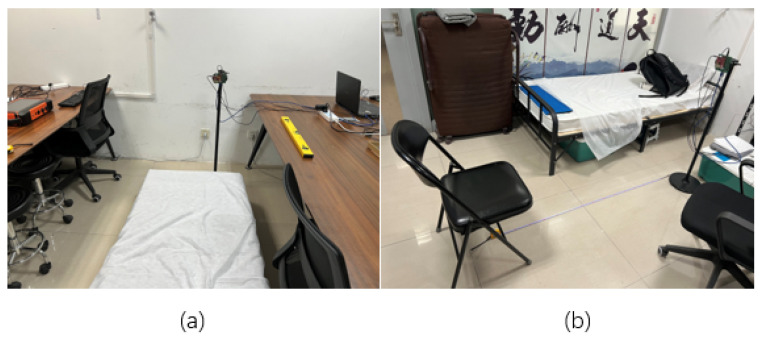
The two uncontrolled indoor environments used for radar data collection. (**a**) The ‘bed scene’, where subjects performed activities while lying on a mattress placed on the floor. (**b**) The ‘sitting scene’, where subjects were seated on a chair in a cluttered room containing furniture and other objects. The Chinese calligraphy visible on the wall in scene (**b**) is decorative and unrelated to the experiment. The FMCW radar sensor, mounted on a tripod, is visible in the background of the scene, positioned to monitor the subject.

Radar signal datasets were collected from 15 subjects in two rooms, as illustrated in Figure 8. In the first room, data were collected in a bed scene, while in the second room, data were collected in a sitting scene. As shown in the figure, our experimental environment was uncontrolled, containing multiple tables, chairs, and other objects, thereby representing a challenging scenario for radar-based monitoring.

For the bed scene, the radar was positioned about 96 cm above the ground and placed about 26 cm horizontally from the bed edge. It was oriented toward the upper part of the bed to primarily capture the subject’s body. Data were collected with subjects either facing the radar directly or at a diagonal angle, and in four postures: supine, left lateral, right lateral, and prone. The bed scene dataset comprises five activity categories: coughing, normal breathing, moving arms, turning over, and sitting up or lying down.

For the sitting scene, the radar was positioned about 94 cm above the ground and oriented horizontally. Data were collected at subject-to-radar distances of about 1 m and 1.5 m. Recordings were performed with subjects facing the radar directly, at a 45° angle, and perpendicular to the radar. The sitting scene dataset comprises five activity categories: coughing, normal breathing, moving arm, moving head, and standing up or sitting down.

Each activity type captures typical movements in the respective scenario. No constraints were imposed on the subjects’ movements to ensure diversity and realism. In addition to coughing and normal breathing, complex actions within each category (e.g., raising hands or scratching the head) were included to enhance the representativeness and practical relevance of the dataset, which also places higher demands on model generalization.

Each data sample was extracted from a 3-second segment. In total, 3165 samples were collected. For the bed scene, 21 cough samples and 82 non-cough samples per subject were recorded, resulting in 1545 samples across 15 subjects. For the sitting scene, 18 cough samples and 90 non-cough samples per subject were recorded, totaling 1620 samples for 15 subjects.

### 3.5. Model Setup and Training Details

As introduced previously, the input is an RDM sequence denoted as $X_{g}^{1,2,\ldots ,T}\in R^{35\times 1\times 48\times 125}$ , where 35 represents the sequence length *T*, and each frame corresponds to a single channel 48×125 feature map. The spatial submodule employs modified ResNet-34 for spatial feature extraction, with two key modifications: adjusting the input channel from 3 to 1, and removing the final fully connected layer. This processing yields spatial feature maps of shape $R^{35\times 512}$ , which are then projected via a linear layer to $f_{g}^{1,2,\ldots ,T}\in R^{35\times 256}$ (the input to the temporal submodule). The temporal submodule adopts a two-layer Transformer-inspired architecture, where each layer integrates multi-head attention with a hidden dimension of 256 and four attention heads. Its output consists of temporal feature maps denoted as $h_{g}^{1,2,\ldots ,T}\in R^{35\times 256}$ , which are then aggregated by average pooling along the temporal dimension to yield a pooled feature representation $k_{g}\in R^{256}$ . Finally, the linear classifier processes $k_{g}$ and maps it to a logit vector $p_{g}^{0,1}\in R^{2}$ , corresponding to the two target activity categories (i.e., cough and non-cough).

For data augmentation, image translation is applied with a maximum displacement of 12 pixels along the distance dimension. Random erasing is further employed by removing a region covering 5–20% of the RDM area, where the width-to-height ratio of the erased region is randomly sampled within the range of 0.3 to 3.33. To preserve temporal consistency, the same augmentation is applied uniformly across all frames within an RDM sequence.

Model performance is evaluated using F1-score and overall accuracy. Five-fold cross-validation is conducted as follows: three non-overlapping subjects are randomly selected from the 15 participants as the test set, and from the remaining 12 participants, 80% of the data are assigned to the training set and 20% to the validation set. The model is trained on the training set, and the parameters achieving the highest F1-score on the validation set are used for testing. Final results are obtained by averaging the performance metrics across the five folds. To ensure representative feature distributions and avoid sampling bias, stratified sampling is applied during dataset partitioning.

The model is implemented in PyTorch (version 2.5.1, running on CUDA 12.1) [35] and trained on a workstation equipped with a 12-core Intel(R) Xeon(R) Silver 4214R CPU (Intel Corporation, Santa Clara, CA, USA), an NVIDIA GeForce RTX 3080Ti GPU (NVIDIA Corporation, Santa Clara, CA, USA) with 12 GB memory, and 90 GB of RAM. The network parameters are optimized using the Adam optimizer with a batch size of 32, a learning rate of 0.0001, a weight decay of 0.3, and a total of 15 training epochs.

## 4. Results

This section presents the experimental results to evaluate the proposed ST-CoughNet. We first report the overall performance of our approach in comparison with baseline methods. Then, we conduct ablation studies to assess the contributions of model components and data augmentation strategies. Finally, we examine the robustness of the proposed method under challenging conditions, including longer distances and novel scenarios. Together, these analyses provide a comprehensive understanding of the effectiveness and generalizability of our model.

### 4.1. Overall Performance

**Figure 9:**
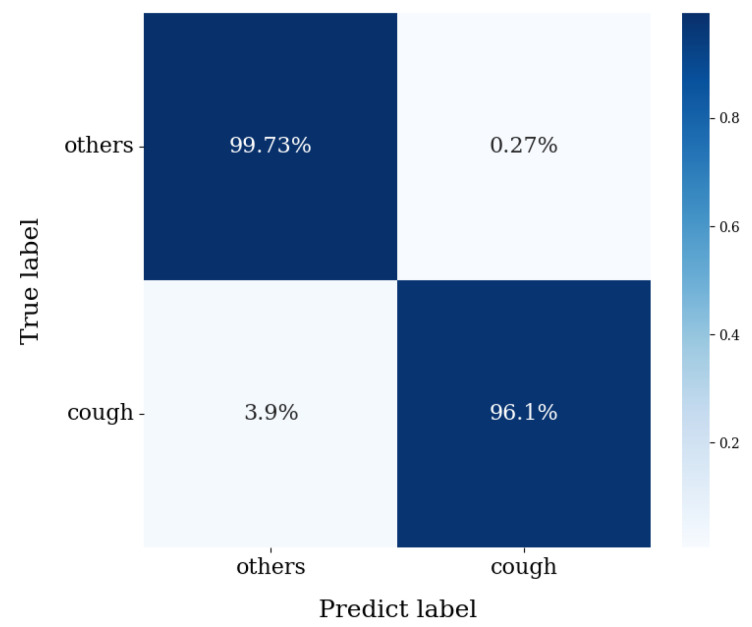
Confusion matrix of the proposed ST-CoughNet under the subject-independent evaluation. The matrix is row-normalized, showing the percentage of samples for each true class that were assigned to a predicted class. The Y-axis represents the true labels, and the X-axis represents the predicted labels.

We first evaluate the overall classification performance of ST-CoughNet under both subject-independent and non-subject-independent conditions, and compare it with representative radar-based baselines. In addition, we further implemented a Transformer model as a stronger temporal baseline, which achieved an F1-score of 0.913 and an overall accuracy of 96.87%. Table 2 summarizes the performance of the proposed method in comparison with existing approaches. The spatiotemporal feature extraction model demonstrates improved performance under a comprehensive dataset and subject-independent evaluation, achieving an F1-score of 0.974 and an overall accuracy of 99.05%. These results suggest that fully leveraging spatiotemporal features in conjunction with data augmentation can enhance the generalization capability for cough recognition. Figure 9 presents the confusion matrix under the subject-independent condition.

In addition, cross-validation results were obtained under a non-subject-independent setting, where the training, validation, and test sets correspond to 64%, 16%, and 20% of the data, respectively. In this scenario, the model achieved an F1-score of 0.979 and an overall accuracy of 99.21%, indicating that the performance slightly decreases when handling new users, further supporting the robustness and generalization of the approach.

To verify that the performance improvement of our proposed ST-CoughNet is statistically significant, we conducted a two-sided paired t-test against the strongest baseline, the Transformer model. The test was performed on the F1-scores obtained from each of the five folds of our subject-independent cross-validation.

The F1-scores for our ST-CoughNet were [0.978, 0.974, 0.996, 0.957, 0.965], while the scores for the Transformer model were [0.911, 0.871, 0.885, 0.961, 0.948]. The paired *t*-test yielded a *p*-value of 0.033.

Since the *p*-value is below the standard significance level of 0.05, we reject the null hypothesis. This result confirms that the superior performance of our proposed ST-CoughNet model over the Transformer baseline is statistically significant.

To provide a more comprehensive evaluation of our proposed ST-CoughNet under the challenging subject-independent condition, we report a full suite of performance metrics in Table 3. In addition to an overall accuracy of 99.05% and an F1-score of 0.9739, the model achieves a high precision of 98.77%, recall of 96.07%, and specificity of 99.73%. This demonstrates its balanced capability in correctly identifying both cough and non-cough events while maintaining a low false positive rate.

In addition to its discriminative power, we also evaluated the model’s calibration using the Brier score, as reported in Table 3. The model achieved a low Brier score of 0.015, indicating that the predicted probabilities are well-calibrated and reliably reflect the true likelihood of a cough event.

**Figure 10:**
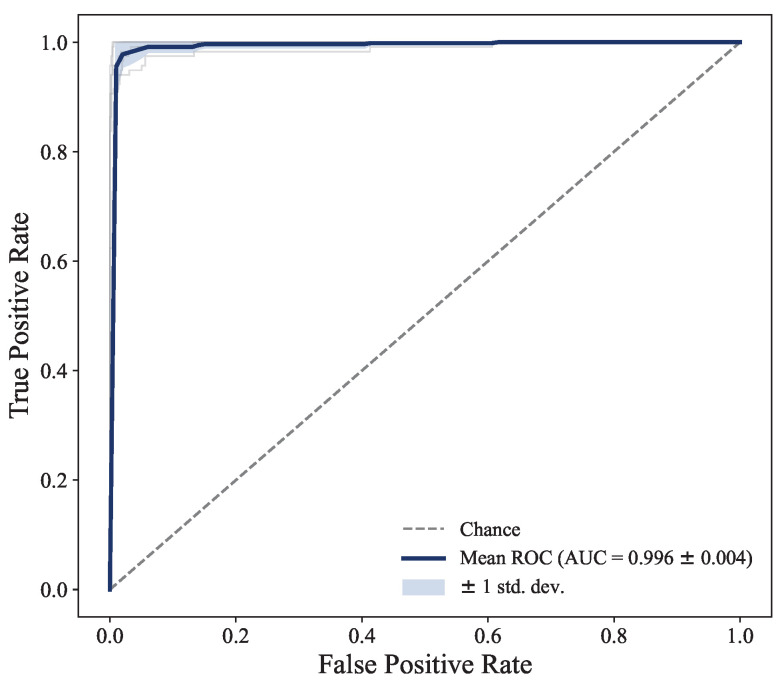
Receiver Operating Characteristic (ROC) curve for the proposed ST-CoughNet model, evaluated under the five-fold cross-validation scheme. The dark blue solid line represents the mean ROC curve interpolated across all five folds. The light blue shaded area indicates the variance (±1 standard deviation) of the curve across the folds. The dashed gray line represents the performance of a random chance classifier (AUC = 0.5) for reference.

Furthermore, the robustness of the model’s classification performance across different decision thresholds is demonstrated by the Receiver Operating Characteristic (ROC) curve in Figure 10. The Area Under the Curve (AUC) reaches an impressive value of 0.996. This confirms that the model maintains high sensitivity and specificity regardless of the chosen threshold, making it a highly reliable classifier for this task.

### 4.2. Ablation Study on Model Components

To clarify the contribution of each submodule, we performed an ablation study by separately evaluating the spatial (ResNet) and temporal (Self-Attention) components, as well as their combination in the full architecture. The model performance was evaluated under three configurations: using only ResNet, using only Self-Attention, and using the ST-CoughNet architecture. Results are summarized in Table 4.

The combined ResNet and Self-Attention model outperforms the individual components. Using only ResNet achieved an F1-score of 0.970 and an overall accuracy of 98.89%, while using only Self-Attention yielded an F1-score of 0.917 and an overall accuracy of 96.90%. The integrated approach achieved an F1-score of 0.974 and an overall accuracy of 99.05%, indicating that jointly capturing spatial features and temporal dependencies provides more comprehensive information for cough recognition.

### 4.3. Ablation Study on RDM Size and Sequence Length

To further assess the robustness of the proposed framework, we conducted additional ablation studies by varying the temporal sequence length and the range-bin resolution of the input RDMs. Specifically, the original configuration ( 35×1×48×125 ) was compared with shorter temporal sequences (first 20 frames and last 20 frames) as well as with modified range-bin dimensions (36 and 60 bins). The results are summarized in Table 5.

The results show that reducing the sequence length leads to a moderate drop in performance, with the first 20 frames yielding an F1-score of 0.943 (accuracy 97.88%) and the last 20 frames yielding 0.904 (accuracy 96.52%). In contrast, changing the range-bin resolution from 48 to either 36 or 60 bins had little negative impact. In fact, both variants maintained high accuracy, with F1-scores of 0.957 (accuracy 98.45%) and 0.965 (accuracy 98.74%), respectively. These findings indicate that ST-CoughNet is robust to variations in spatial resolution and sequence length, although sufficient temporal context remains important for optimal performance.

### 4.4. Ablation Study on Data Augmentation

Next, we investigate the effect of different radar-aware augmentation strategies to determine whether they improve the robustness and generalization of the proposed method. The effect of data augmentation was evaluated under four conditions: without augmentation, using only random erasing, using only image translation, and applying both methods simultaneously.

To provide a granular, per-class analysis of the trade-offs involved, the detailed performance metrics for each strategy are summarized in Table 6.

The results demonstrate that data augmentation consistently improves overall performance, with the combination of both methods yielding the best results. Without any augmentation, the model achieved an F1-score of 0.953. While individual techniques like translation increased the F1-score to 0.964, they achieved so by improving precision and recall for the ‘cough’ class.

Crucially, the combined strategy of Random Erasing + Translation provided the most significant and balanced improvement. It achieved the highest F1-score (0.974) and accuracy (99.05%), driven by a substantial increase in both precision (97.10%) and recall (95.10%) for the ‘cough’ class. This indicates that the synergy between the two augmentation methods facilitates learning richer and more generalizable representations, leading to a more robust and effective cough recognition model.

### 4.5. Performance at Longer Distances

To assess robustness to variations in target distance, data collected at closer distances were used for training and validation, while data from farther distances were reserved for testing. For the bed scenario, subjects facing the radar were used for training/validation, and diagonally oriented subjects for testing. For the seated scenario, subjects at 1 m were used for training/validation and at 1.5 m for testing. Results are presented in Table 7.

The proposed method achieved an F1-score of 0.954 and an overall accuracy of 98.3%. Compared with using only ResNet or Self-Attention, the performance decrease is minimal, suggesting that the model is robust to variations in subject position.

### 4.6. Performance in Novel and Per-Scene Scenarios

To further evaluate robustness to environmental variations, we analyzed the model’s performance separately for different scenes (bed vs. seated). Specifically, training and validation were performed in one scenario, while testing was conducted in the other, with a training-to-validation ratio of 4:1. This design enables a per-scene evaluation and examines the generalization ability of the proposed method across distinct environments. Results are summarized in Table 8.

The proposed method consistently outperforms models using only ResNet or Self-Attention in both bed and seated scenarios. When tested on the seated scenario, the F1-score and overall accuracy were 0.836 and 93.41%, respectively. When tested on the bed scenario, they reached 0.989 and 99.55%. The performance decrease in the seated scenario can be attributed to weaker signal strength and more diverse body movements, highlighting the increased complexity of the data. Despite these challenges, the method demonstrates strong performance in novel scenarios, indicating robustness to environmental variations.

## 5. Discussion and Conclusions

In this study, we proposed ST-CoughNet, a deep learning-based spatiotemporal feature extraction framework for FMCW radar-based cough recognition. By integrating ResNet for spatial feature extraction and a Self-Attention mechanism for temporal modeling, the framework effectively captures both the range–velocity patterns within individual RDMs and the temporal dynamics across the entire signal sequence. Two tailored data augmentation strategies—image translation and random erasing—were shown to further enhance the model’s generalization capabilities.

The experimental results demonstrate that ST-CoughNet achieves high accuracy and robust performance across different subjects and conditions, with its superiority over baseline models confirmed to be statistically significant. Compared to previous radar-based cough recognition studies [20,21], our method not only attains superior recognition metrics but also explicitly models temporal evolution, which is often neglected in earlier approaches.

Despite these promising results, a qualitative analysis of the misclassified cases reveals specific limitations and provides valuable insights. Our error analysis indicates that misclassifications primarily fall into two categories. False Positives, where non-cough events were mistaken for coughs, were typically caused by other abrupt, short-duration motions that generate strong, transient micro-Doppler signatures visually similar to a cough in the RDM. Actions such as a sudden torso shift or head movement were the main sources of this confusion, as they can create a vertical stripe pattern in the RDM that mimics a true cough event. Conversely, False Negatives, where coughs were missed, were predominantly associated with very weak or atypical coughs. These events often produced radar signatures with an insufficient signal-to-noise ratio, making their patterns in the RDM faint and difficult to distinguish from background noise.

These findings directly inform avenues for future work. The confusion between coughs and other explosive bodily motions highlights the need for more advanced feature extraction techniques capable of distinguishing these fine-grained spatiotemporal differences. Future research could explore attention mechanisms with higher temporal resolution or multi-scale feature fusion to better capture the subtle characteristics unique to a cough. Furthermore, to address the challenge of weak signals, adaptive preprocessing and signal enhancement algorithms could be investigated to improve performance in noisy or distant scenarios.

In conclusion, ST-CoughNet demonstrates notable potential for healthcare applications. Its contactless and privacy-preserving design enables continuous cough monitoring in home or clinical settings, supporting the early detection and management of respiratory conditions. Beyond cough recognition, the proposed spatiotemporal framework provides a versatile and robust foundation for other radar-based respiratory and activity monitoring tasks in the field of bioengineering.

## Figures and Tables

**Table 1:** Radar configuration parameters.

Parameter	Value	Unit
Start Frequency	60	GHz
Bandwidth	3.96	GHz
Slope	60.011	MHz/μs
Idle Time	300	μs
TX Start Time	1	μs
ADC Start Time	6	μs
Sample Rate	2000	kS/s
Ramp End Time	60	μs
Chirp Repetition Frequency	500	Hz
Chirp Number	1500	–
ADC Samples per Chirp	108	–
Number of Transmitters	1	–
Number of Receivers	4	–
Range Resolution	0.042	m
Velocity Resolution	0.008	m/s

**Table 2:** Comparison of the proposed method with existing approaches.

Method	Radar	Task (Dataset Composition)	Independent Subjects	F1-Score	Overall Accuracy (%)
AlexNet [17]	Doppler	5 Total Activities	No	-	86.50
MPC + CNN [18]	FMCW	3 Total Activities	No	0.290	71.00
Transformer	FMCW	Binary (1 Cough + 6 Non-Cough)	Yes	0.913	96.87
Ours	FMCW	Binary (1 Cough + 6 Non-Cough)	Yes	0.974	99.05
Ours	FMCW	Binary (1 Cough + 6 Non-Cough)	No	0.979	99.21

**Table 3:** Detailed performance metrics of the proposed ST-CoughNet.

Metric	Value (Mean ± Std Dev)
Accuracy (%)	99.05±0.55
Precision (%)	98.77±1.05
Recall (Sensitivity)(%)	96.07±2.16
Specificity (%)	99.73±0.23
F1-score	0.974±0.016
Brier Score	0.015±0.012
AUC	0.996±0.004

**Table 4:** Performance of different model configurations.

Method	F1-Score	Overall Accuracy (%)
ResNet	0.970	98.89
Self-Attention	0.917	96.90
Ours	0.974	99.05

**Table 5:** Performance under different RDM sequence lengths and range-bin sizes.

Configuration	Sequence Size	F1-Score	Overall Accuracy (%)
Original	35×1×48×125	0.974	99.05
First 20 frames	20×1×48×125	0.943	97.88
Last 20 frames	20×1×48×125	0.904	96.52
Reduced range-bin	35×1×36×125	0.957	98.45
Increased range-bin	35×1×60×125	0.965	98.74

**Table 6:** Model performance with various data augmentation strategies.

Data Augmentation	F1-Score	Accuracy (%)	Precision (%)	Recall (%)	Specificity (%)
None	0.953	96.36	94.60	91.70	99.20
Only Random Erasing	0.959	98.52	95.40	92.80	99.40
Only Translation	0.964	98.67	96.00	94.00	99.50
Random Erasing + Translation	0.974	99.05	97.10	95.10	99.60

**Table 7:** Performance for predicting distant data using proximal data.

Method	F1-Score	Overall Accuracy (%)
ResNet	0.929	97.33
Self-Attention	0.855	95.14
Ours	0.934	97.62

**Table 8:** Per-scene performance (bed vs. seated) and generalization to novel scenarios.

Method	Test Scene	F1-Score	Overall Accuracy (%)
ResNet	Seated	0.802	92.01
Self-Attention	Seated	0.727	88.95
Ours	Seated	0.836	93.41
ResNet	Bed	0.936	97.18
Self-Attention	Bed	0.773	92.54
Ours	Bed	0.989	99.55

## Data Availability

The data presented in this study are available on request from the corresponding author due to the protection of participants’ privacy.

## Abbreviations

The following abbreviations are used in this manuscript:

FMCW	frequency-modulated continuous wave
CNN	convolutional neural network
RX	received signal
TX	transmitted signal
IF	intermediate frequency
FFT	Fast Fourier Transform
RDM	range-Doppler map
